# Young People’s Views on Electronic Mental Health Assessment: Prefer to Type than Talk?

**DOI:** 10.1007/s10826-014-9929-0

**Published:** 2014-02-26

**Authors:** Sally Bradford, Debra Rickwood

**Affiliations:** Faculty of Health, University of Canberra, Building 12, D27, Canberra, ACT Australia

**Keywords:** Electronic assessment, Self-disclosure, Mental health, Youth, Psychosocial

## Abstract

For mental health professionals to provide personalized early interventions, young people need to disclose sensitive information to a clinician they are unlikely to have yet formed a relationship with. We conducted in-depth qualitative interviews with 129 young people aged 12–25 years from several sites across Australia to gauge views on whether young people thought that an electronic psychosocial assessment tool could help them initially disclose personal information. Additionally, we were interested in whether young people from different demographic groups held similar views around using the e-tool. Results provided support for the use of an e-tool, with most young people stating that it could help in the disclosure of particularly embarrassing problems. The main advantages reported were that the e-tool would support disclosure without fear of judgment by health professionals, and would enable young people greater input in deciding what to focus on. Young people who held a preference to simply talk were most concerned about the clinician missing non-verbal cues. These findings highlight the value of incorporating electronic options within clinical practice, but also the need for health professionals to work within a flexible framework guided by the individual preferences of each of their clients.

## Introduction

The majority of mental disorders emerge in adolescence and young adulthood (Belfer 2008; de Girolamo et al. 2012), with three-quarters of all lifetime cases occurring before 24 years of age (Kessler et al. 2005). Mental health problems in young people significantly impact on their social, emotional, physical, and educational development (Kazdin 1993; Strauss et al. 1987), and are likely to continue well into adulthood if left untreated (Catania et al. 2011; de Girolamo et al. 2012). Fortunately, early intervention and prevention strategies can substantially improve the outlook (McGorry et al. 2011) but, in order for mental health professionals to provide appropriate intervention, assessments that give an accurate and holistic picture of the young person and their life are required (Leavey et al. 2008). These assessments need to cover multiple social, emotional, and behavioral domains relevant to the young person’s wellbeing (Bradford and Rickwood 2012). However, such assessments rely on the young person feeling comfortable enough to disclose their highly personal issues to a relative stranger. Helping young people to disclose what is happening for them early in the treatment process will enable mental health professionals to work collaboratively with the young person to provide a holistic and personalized treatment plan.

Within counselling and clinical mental health care, mental health professionals need to obtain a holistic assessment of their clients within the first or second session, so that an appropriate treatment plan can be developed. To help young people through the difficult process of self-disclosing intimate and personal information, mental health professionals can use specific psychosocial assessments. The ‘HEEADSSS assessment’, which stands for **H**ome, **E**ducation/employment, **E**ating, **A**ctivities and peer relations, **D**rugs and Alcohol, **S**exuality, **S**uicide/depression, and **S**afety, is a common assessment used throughout the United States that structures questions to maximize communication and minimize stress on the young person (Cohen et al. 1991; Goldenring and Rosen 2004). The order of the domains specifically leads the young person from the less personal domains of home life and school, through to the highly personal domains of sexuality and suicide. The domains covered by the HEEADSSS assessment are representative of the domains covered by many psychosocial assessments (Bradford and Rickwood 2012) and are commonly assessed as they reveal many of the risk and protective factors affecting mental health in young people (Cohen et al. 1991). For example, young people with emerging issues regarding their sexuality, are homeless, or are using alcohol or other illicit drugs are significantly more likely to be dealing with mental health problems (AHRC 2012; King et al. 2008; Mackesy-Amiti et al. 2012). While it is important to ask young people whether they have such demographic risk factors, the stigma surrounding issues such as sexuality, homelessness, and substance use, may hinder initial help-seeking and early disclosure (Corrigan and Rao 2012; Eisenberg et al. 2009).

The majority of psychosocial assessments take the form of a self-administered questionnaire (Harrison et al. 2001) or a semi-structured interview format (Goldenring and Cohen 1988; Parker et al. 2010). A recent systematic review of psychosocial assessment tools available for use with the general population of young people aged 12–25 years, found that young people were generally more accepting of assessments that were initially self-administered through a questionnaire, rather than those that relied completely on verbal disclosure to a clinician (Bradford and Rickwood 2012). The preference for a self-administered questionnaire may be due to the young person having increased feelings of control in the disclosure process as they have the time to organize their thoughts and feelings. For example, a study by Elliott et al. (2004), found that the implementation of an adolescent intake questionnaire allowed adolescents to identify the issues that were of most importance to them, identify the domains they were ready to discuss immediately and those that would need to be returned to, signaled that it was okay to disclose certain information, and helped them to structure their thoughts by providing a time for personal reflection.

While there appears to be support for the use of self-administered psychosocial assessments over those that rely entirely on verbal disclosure, it is unclear whether computer administered assessments result in an increase in disclosure rates over those that are completed in a pen-and-paper format (Bradford and Rickwood 2012). The mixed research findings are interesting considering the commonly held assumption that young people disclose more personal information in online modalities due to the online disinhibition effect (Suler 2004; Wallace 2001). It should be noted, however, that the studies comparing pen-and-paper and computer administered psychosocial assessments have all used large desk top computers, which were often situated within waiting rooms (Beebe et al. 2006; Raat et al. 2007; Silber and Rosenthal 1986; Truman et al. 2003). In this format, it is likely that young people did not feel that their information was particularly private—an important aspect for young people seeking help for their mental health problems (Bradley et al. 2012). New tablet device technology, which allows young people to complete assessments on a smaller screen, may provide a greater sense of privacy. With personal tablet devices now outselling PC laptops (NPD 2013), and projected to outsell PC laptops and PC desktops combined by 2017 (Milansesi et al. 2013), this new tablet technology deserves specific investigation. There is also no research investigating how young people from different demographic groups, particularly those with strong mental health risk factors, respond to different types of assessment formats.

If early intervention is to be effective, it requires young people to feel confident disclosing highly personal information at an early stage of service engagement. This may be facilitated through self-administered assessments using tablet technology which allows young people to disclose privately in a less intimidating format. As such, the aim of the current research was to identify whether young people felt that an electronic psychosocial assessment tool (e-tool) on a tablet device could improve rates of initial disclosure of sensitive issues to mental health professionals within the standard face-to-face therapy format. We were also interested in whether these views differed for the various psychosocial domains assessed. Additionally, we investigated whether specific views were related to the age of the young person or their identification to relevant higher-risk demographic groups, including those who identified as lesbian, gay, bisexual, transgender or intersex (LGBTI), were homeless, had alcohol or other drug (AoD) problems, had previously used mental health services, or came from an Indigenous background.

## Method

### Participants

Participants comprised a total of 129 young people aged 12–25 years from two major cities in Australia—Canberra in the Australian Capital Territory, and Melbourne, Victoria. Just over half the participants were females (57 %) and 43 % were males. Participants were drawn from specific demographic groups of interest, as shown in Table 1. This included young people from the general community (*n* = 54), attending a mental health service (*n* = 39), identified as being LGBTI, homeless, Indigenous, or were involved in an AoD service. The community and mental health groups were conducted within the following age groups: 12–14, 15–17, 18–21, and 22–25 years.

**Table 1:** Participant demographics of each interview group

Group type	Age range (years)	Boys	Girls	Total
Community 12–14 years	12–14	6	12	18
Canberra	12–14	2	7	9
Melbourne	12–14	4	5	9
Community 15–17 years	15–17	8	15	23
Canberra	15–17	8	13	21
Melbourne	15–17	0	2	2
Community 18–21 years. Canberra	19–21	6	2	8
Community 22–25 years. Canberra	22–25	3	2	5
Youth mental health service 12–14 years. Canberra	12–14	2	3	5
Youth mental health service 15–17 years. Canberra	15–17	2	3	5
Youth mental health service 18–21 years. Canberra	18–20	2	4	6
Youth mental health service 22–25 years	22–25	8	15	23
Canberra	22–25	8	11	19
Melbourne	22–23	0	4	4
Alcohol and other drug. Canberra	15–18	4	3	7
Indigenous. Canberra	13–18	5	4	9
Homeless	15–18	3	7	10
Canberra	15–18	2	4	6
Melbourne	15–17	1	3	4
Lesbian, gay, bisexual transgender, intersex	16–23	7	3	10
Canberra	16–23	5	2	7
Melbourne	17–23	2	1	3
Total	12–25	56	73	129

### Procedure

Prior to the study commencing, ethics approval was obtained from the University of Canberra Committee for Ethics in Human Research (Approval no. 12-125). The study was advertised through schools, relevant community organizations, and mental health services, with interested participants contacting the research team. Participants were organized into interview groups based on their identification with the demographic categories of interest. Initially, all groups were formed from Canberra residents; however, to ensure responses were not specific to the Canberra region, some matched groups were also held in Melbourne, Victoria (see Table 1). Participant recruitment and interviewing was run from September 2012 through to February 2013 and ceased when there was adequate numbers across age and demographic groups, and saturation of key themes was achieved (Guest et al. 2006).

At the beginning of each group interview, participants were informed of the purpose of the study and that it would be audio recorded and transcribed verbatim. All participants signed a consent form, and participants aged 14 years or younger also required signed parental consent. Interviews ranged in length from 16 to 49 min (*M* = 32.75, *SD* = 9.31) and participants received two movie tickets or a A$25 gift voucher for participating. Three of the 26 group interviews were facilitated by The Australian Foundation for Mental Health Research (AFFIRM) Youth Ambassadors. This participatory research design was employed firstly as part of an overall commitment to genuine youth participation in the research protocol (AICAFMHA 2008), and also to ensure that group responses were not affected by the age of the primary facilitator, and to further ensure that appropriate language was used within the interview guide (Delman 2012; Kidd and Kral 2005). After these initial three groups had been conducted and the interview guide altered accordingly, the remaining groups were facilitated by the first author.

### Interview Guide

The interview guide was developed in conjunction with input from members of the headspace Youth National Reference Group (hY NRG). This was another design component incorporated to ensure that a youth participation framework underpinned the research (AICAFMHA 2008). Along with the AFFIRM Youth Ambassadors, the hY NRG members ensured the interview questions and procedure were youth-appropriate.

In the final interview guide, participants were first told about the process they could expect to encounter when attending a service for mental health care and how a psychosocial assessment e-tool would be incorporated if it were to be developed. Participants were then asked the following open-ended questions to prompt discussion specifically about the e-tool: When you first go to a counselor or psychologist would you find it easier telling them stuff about yourself using an assessment e-tool or speaking? Or is it the same? Do you feel the same for all of the different parts of your life or would some be easier disclosing on an assessment e-tool rather than face-to-face? Or vice versa?

### Analytic Strategy

Interviews were transcribed verbatim and analyzed with the aid of NVivo 10 software (QSR 2012). A critical realist, thematic analysis approach was utilized, with the data being analyzed at a semantic level (Braun and Clarke 2006; Miles and Huberman 1994). Specifically, the thematic coding was conducted using a hybrid approach of deductive coding and inductive coding, which has been demonstrated as resulting in rigor within qualitative research designs (Fereday 2006). Initially, a deductive approach was taken, whereby domain codes were developed based on an a priori template of the questions asked within the interview guide (Crabtree and Miller 1999). An inductive thematic approach was then used to code all participant responses.

Each interview was systematically coded by the first author in a data driven manner, so that codes were created to cover all parts of the data (Richards and Morse 2007; Thomas 2006). Over several meetings between both authors, a thematic map was created to organize the codes into themes and sub-themes under the domains created by the a priori template (Braun and Clarke 2006; Fereday 2006). To determine the level of representativeness of responses, four levels of frequency labels were applied. As proposed by Hill et al. (2005), a theme that applied to all or all but one of the cases was considered *general*. A *typical* theme applied to more than half of the cases. A theme considered *variant* included at least three cases and up to half of all cases. A theme that included two or three cases was considered *rare*. Findings that emerged from only one case were not reported.

To provide additional support for the reliability of the analysis, an external auditor recoded 15 % of the data (Guest and MacQueen 2008; Hill et al. 2005). Inter-rater reliability was assessed using combined segment-based Cohen’s Kappa scores on two double-coded transcripts (Carey et al. 1996). NVivo 10 software computes Cohen’s Kappa by calculating the percentage of agreement and disagreement between raters while taking into account the amount of agreement that could be expected to occur through chance (QSR 2012). The averaged Cohen’s Kappa score for all codes and recoded-sources was .80, indicating excellent inter-rater reliability (Cohen 1960; Guest et al. 2006).

## Results

### Views on Using an Assessment E-tool

Responses to the question of whether it would be easier telling a counselor or psychologist about themselves using an e-tool or speaking in person, revealed two general themes labeled ‘Prefer to initially type’ and ‘Want to talk’.

#### Prefer to Initially Type

Under the theme ‘Prefer to initially type’, sub-themes were identified as reasons why young people believed the e-tool would make initial disclosure easier. Two typical themes were identified: the ability to disclose without the fear of any initial judgmental reactions from clinicians (*No judgmental reactions*) and *providing a structure to thoughts and sessions.* Young people stated that they would be “more comfortable being able to just write it down and then not having to say it” (Female, Community 15–17 years) and “talking to a computer screen makes it so much easier and you just don’t think about what the computer screen is going to think of you” (Female, Community 15–17 years). Under the theme *provide a structure to thoughts and sessions* comments focused around the young person being able to pinpoint the areas they want to focus on and indicate to the clinician the areas that should be probed further. One participant made the comment that being able to answer questions prior to seeing the clinician face-to-face would give them a “heads up” (Male, Community 12–14 years). Another participant felt that it would allow herself to build the courage needed to disclose her thoughts:

So giving them that time to - because someone might ask you a question and you know you’re battling on the inside, like about to answer, like working up the nerve to be able to answer and tell them something and then if they say oh, okay, it’s all right - just being able to have that silence to…Kind of adjust. (Female, Community, 15–17 years)

Other participants noted the benefit in terms of indicating the important issues to the clinician so those could be of focus within session. For example, one participant stated “as then you don’t have that awkward moment when they keep asking about this one thing, and you’re like, that’s not exactly what I wanted to talk to you about, but sure” (Female, Youth Mental Health group, 18–21 years). And another participant noted:

I find that it’s easier for them to already know a tiny bit about you and that helps that process because I’m very nervous starting to talk about my problems and so if they already know a bit and they can just ask me for more information, I feel like that helps me build a personal rapport with them. (Female, Youth Mental Health group, 15–17 years)

Four variant themes were also identified. These included the belief that using the e-tool would allow *disclosure to occur in a stepped process*, *save time,* as “That way you have a little more time to actually get to the problems rather than trying to fill in the basics and stuff” (Female, Youth Mental Health group, 15–17 years), allow young people to disclose in a modality they are comfortable with (*comfortable with the online modality*), and allow some young people the opportunity to better articulate their concerns (*more articulate when writing*) as “some young people can’t articulate themselves in person very well so having it written … could be easier for people to understand how you’re feeling” (Male, Youth Mental Health Service, 22–25 years). Additionally, a rare theme was stated by two different groups, who felt that the e-tool would provide health professionals with the *necessary background information without* [young people] *becoming overwhelmed by emotions*.

#### Want to Talk

Most young people also provided reasons to want to talk, although this was the primary preference for only a sub-sample of the participants. The only typical reason why young people stated that they ‘Want to talk’ focused around the *importance of non*-*verbal language.* For example, a female participant from the homeless demographic group made the comment that, “On the computer when you’re typing and stuff, they can’t actually see your body language and they can’t really tell how upset you actually are”. The essence of this sub-theme emphasized the belief that some young people may be in significantly more distress than they indicate, which may be missed if the e-tool is the primary source of information.

Three variant sub-themes were also identified: a concern for *privacy and confidentiality* if their information was ‘hacked’; the belief that *talking provides more in*-*depth responses* because “a lot of surveys, they give you scales or they don’t give you enough to give enough information for what you want to say” (Female, LGBTI group); and the concern that *questions may be misinterpreted* and therefore answered incorrectly. A rare theme was also stated by two groups who felt that there was a sense of permanence when issues are put into writing that is not felt when you talk to a health professional (*writing is permanent*).

### Domain Specific Views

In order to determine whether the assessment e-tool would be appropriate for use with all the relevant psychosocial assessment domains, we asked young people whether they felt that using the assessment e-tool would be easier for certain domains or whether they felt that for some domains it would not be appropriate. Overall, the most common response was that participants felt that the more embarrassing domains would be *easier* to disclose using a tablet device. For example, one participant stated:

I would say that all of those would be easier on an iPad, because you don’t have to admit anything to someone and it would be like saying you’re depressed, or saying you’re gay, or saying whatever. It would be easier to say it on a - just put it on a computer than to say it to some random guy. (Male, Community, 12–14 years)

### Age Group Differences

To investigate whether views on using the e-tool were related to age, group cases were created within NVivo 10 so that group responses were combined for all young people aged 12–14, 15–17, 18–21, and 22–25 years. The ‘framework matrix’ function of NVivo 10 was then used to compare responses within each theme and sub-theme across the four age groups. The general theme that they would ‘Prefer to initially type’, was noted by participants across all age groups; however, the age groups who appeared to most strongly identify with this theme were those in the 15–17 and the 18–21 year age groups, who made a number of comments around the usefulness of the tool in *providing a structure to thoughts and session, disclosing in a stepped process, being comfortable in the online modality,* and *providing the background information without getting emotional.* Comments made from these groups included:

…that’s kind of like what they do when you go into chat online or Kids Helpline. You say, I’m - it’s just a basic I’m interested in this, I live with my parents and that kind of thing, my name is this or whatever. That’s like you don’t even really think twice when you put the dots in or tick the boxes but it makes it a lot easier. (Female, Youth mental health service, 18–21 years)

It’s a good first step. It’s a good first step just to get the information out and then you can go the second step where you can meet up and they already know the actual information. (Male, Community, 15–17 years)

…most people who go to the first initial consultation…don’t always want to talk about that to a person that they barely even know. So I reckon that would be a good way to start the ball rolling really so we don’t go through all the - like they already have all the answers there. (Female, Youth mental health service, 18–21 years)

The theme ‘Want to talk’ was also identified by all age groups, but most strongly by those participants aged 22–25 years who highlighted the *importance of non*-*verbal information*, the ability to *provide more in*-*depth responses when talking* and concerns around disclosing illegal behaviors on a permanent record:

If you were filling out a form on a tablet, it’s just your age, your address. Whereas if you’re face-to-face, oh, where do you live. Oh where’s that? It’s open-ended questions. (Female, Community, 22–25 years)

I guess the other thing - if you’re talking about drugs and alcohol or any kind of illegal activity or anything, you were writing down that you’ve done it. Then there’s that evidence or whatever that you’ve said this. (Female, Community, 22–25 years)

If you ask me then you would see me stuttering my answer. On an iPad you wouldn’t see any of that. So you just ignore the issue…so face-to-face is maybe still a better idea after all so they know how they’re feeling cause they can see the expressions on your face… (Male, Community, 22–25 years)

Young people in the 12–14 years groups did not appear to hold stronger views for either of the general themes, stating that they thought the e-tool would help them to know what to expect in the session and would allow the clinician to “get an idea of what your actual problems are” (Female, Youth mental health, 12–14 years), but they were also concerned about the *privacy and confidentiality* of their information.

### Demographic Group Differences

To determine whether views on using the e-tool were related to demographic group type, group cases were created within NVivo 10 so that group responses were combined for young people: within the general community; attending a mental health service; who were homeless; identified as having AoD issues; were Indigenous; or identified as LGBTI. The ‘framework matrix’ function of NVivo 10 revealed differences for the Indigenous and homeless participant groups. The Indigenous participants appeared to strongly identify with the general theme ‘Prefer to initially type’ stating the typical themes of *no judgmental reactions* and *provides a structure to thoughts and session.* This group did not make any comments around preferring to talk. A male Indigenous participant stated “it comes from the whole barrier of being shy. So yeah it just cuts off the whole facial expression or those looks you get, so that you don’t feel that they judge you”. An Indigenous female participant commented, “If you do answer those on the iPad they know where you’re at, and I guess pinpoint areas that you need to talk about.” Conversely, the homeless participants focused more on why they ‘Want to talk’. This group of participants focused primarily on the importance of non-verbal communication:

If you had someone in front of you, you’re able to understand a lot more about them just through their body language and what-not. So like if you were to be writing something down, I reckon someone’s more likely to lie through that. (Female, Homeless, 15–18 years)

## Discussion

The aim of the current research was to investigate how young people feel about the use of an electronic psychosocial assessment tool, using small tablet device technology, and whether it would be a barrier or facilitator to initial disclosure of sensitive issues to mental health professionals. In general, most participants felt that the e-tool would be particularly useful in helping them disclose the domains that they were most embarrassed about, and overall would be open to the idea of using the assessment e-tool to initially indicate concerns in all of the relevant psychosocial domains. Views were similar to the findings of Elliott et al. (2004), in that participants felt that the e-tool would help provide a structure to their thoughts and the overall session by allowing them to identify issues of importance and take the time to decide what they were ready to disclose. These views indicate that young people would like greater input into the mental healthcare process and their discussions within session. Providing young people with increased feelings of involvement in their mental health care may lead to greater patient satisfaction (Swanson et al. 2007) and improved mental health outcomes (Clever et al. 2006) and is in line with the overall move toward shared decision making (SDM) in healthcare (Charles et al. 1997; Simmons et al. 2012). The smaller proportion of young people who felt they ‘Want to talk’, were mostly concerned around the need for non-verbal cues. This stemmed from the belief that some young people may under-report the seriousness of their mental health problems. Interestingly though, the majority of participant groups who had responses categorized under the theme ‘Want to talk’, also had responses categorized as reasons why they felt the e-tool could be of benefit, indicating that even those young people who held a preference to talk, could also see the positive utility of an e-tool.

There were some differences in the willingness to use the e-tool across the different age groups. Participants aged 15–21 years, most strongly identified with the belief that the e-tool would help them initially disclose, while participants aged 22–25 years were more likely to want to just talk. Participants aged 12–14 years did not appear to identify more strongly with either theme. This age trend may be an indication of varying levels of mental health literacy, digital experience, and/or knowledge of traditional health care modes of delivery. The youngest participants probably have the lowest levels of mental health literacy and health care seeking experience (Furnham et al. 2013) and, therefore, are unlikely to have strongly formed views on the subject. Participants aged 15–21 years are more likely to be currently struggling with sensitive issues (Pottick et al. 2008) and therefore more likely to be able to identify with what it would be like to disclose the problem, and how this could be made easier through the use of technology. Finally, it would be expected that those participants aged 22–25 years have greater knowledge and experience in seeking mental health care through the traditional face-to-face method; consequently, they are more likely to prefer to talk as this is the method they are most familiar with.

Some differences were also identified when comparing responses across the demographic groups. The Indigenous group were particularly interested in the use of an e-tool as they felt it would reduce the likelihood of experiencing judgmental reactions from clinicians and help them identify their issues of greatest concern. Considering the well documented history of discrimination and disempowerment of Indigenous communities, and how this continues to affect their mental health and wellbeing (Wexler 2009; Williamson et al. 2010), the comments made by this demographic group are particularly important. If the e-tool can decrease the fear of judgment, and increase the input by Indigenous youth in their treatment experience, we may see significantly greater help-seeking and satisfaction in mental health services by these young people. In contrast, participants who were homeless held a stronger preference to talk, emphasizing the importance of non-verbal cues. These participants were currently staying in a short-term supportive housing environment staffed by professionals working in the traditional face-to-face service model; consequently, like the older participants in the study, their preference may be based upon their familiarity with the face-to-face model. Additionally, this group is likely to be highly aware of the stigma surrounding homelessness and the likelihood of young people attempting to conceal the severity of their current situation (Hudson et al. 2010; Kidd 2007). As this group of participants were currently involved in a homeless support service they may have realized the benefit in seeking help and recognize the need for professionals to use non-verbal cues to identify issues in other young people who may be attempting minimize their problems.

For a qualitative design, this study had a large number of participants recruited from two major metropolitan areas of Australia. Nevertheless, active participation was required on behalf of the participants and the sample is likely to be biased towards those with an interest in improving mental health care for young people. Further, because the interactive e-tool has not yet been developed, understanding the concept required some abstract reasoning abilities on behalf of the participants, and some found this challenging. Finally, due to the already large number of interview groups being run, it was not possible to further split groups by gender, which would have been of interest as gender and age have an interactive effect on mental health help-seeking (Zwaanswijk et al. 2003). These help-seeking effects may also be evident in the willingness to disclose personal issues. This research also only investigated the views of young people and future research needs to determine how mental health professionals would view the use of such a tool. Mental health workers spend a great deal of time on assessment and it is often used as an opportunity to engage with the young person and build rapport. Therefore, their views on such a change to practice also need to be well understood.

Helping young people disclose sensitive issues is vital for mental health professionals in providing early intervention and prevention (Leavey et al. 2008; McGorry et al. 2011). The results of the current study provide support for the use of a psychosocial assessment e-tool within face-to-face mental health care with most young people stating that the e-tool would help in the disclosure of particularly embarrassing problems, and is a preferable method of disclosure as it increases their control over the help-seeking and disclosure process by allowing them to structure their thoughts and indicate areas of most importance. The possibility that an e-tool could provide young people with feelings of greater input into their treatment is an important implication and is in line with the increasingly popular move towards SDM in health care. By working in a flexible manner and providing the option for young people who would prefer to initially disclose by using an e-tool to do so, clinicians will be giving their clients greater choice and input into their mental health care which will likely lead to significantly better patient satisfaction and improved overall health outcomes (Clever et al. 2006; Swanson et al. 2007).
